# Blebbistain, a Myosin II Inhibitor, as a Novel Strategy to Regulate Detrusor Contractility in a Rat Model of Partial Bladder Outlet Obstruction

**DOI:** 10.1371/journal.pone.0025958

**Published:** 2011-10-07

**Authors:** Xinhua Zhang, Allen Seftel, Michael E. DiSanto

**Affiliations:** 1 Department of Urology, Albert Einstein College of Medicine, Bronx, New York, United States of America; 2 Department of Surgery/Division of Urology, Cooper Medical School of Rowan University, Camden, New Jersey, United States of America; Florida International University, United States of America

## Abstract

Partial bladder outlet obstruction (PBOO), a common urologic pathology mostly caused by benign prostatic hyperplasia, can coexist in 40–45% of patients with overactive bladder (OAB) and is associated with detrusor overactivity (DO). PBOO that induces DO results in alteration in bladder myosin II type and isoform composition. Blebbistatin (BLEB) is a myosin II inhibitor we recently demonstrated potently relaxed normal detrusor smooth muscle (SM) and reports suggest varied BLEB efficacy for different SM myosin (SMM) isoforms and/or SMM vs nonmuscle myosin (NMM). We hypothesize BLEB inhibition of myosin II as a novel contraction protein targeted strategy to regulate DO. Using a surgically-induced male rat PBOO model, organ bath contractility, competitive and Real-Time-RT-PCR were performed. It was found that obstructed-bladder weight significantly increased 2.74-fold while *in vitro* contractility of detrusor to various stimuli was impaired ∼50% along with decreased shortening velocity. Obstruction also altered detrusor spontaneous activities with significantly increased amplitude but depressed frequency. PBOO switched bladder from a phasic-type to a more tonic-type SM. Expression of 5’ myosin heavy chain (MHC) alternatively spliced isoform SM-A (associated with tonic-type SM) increased 3-fold while 3’ MHC SM1 and essential light chain isoform MLC_17b_ also exhibited increased relative expression. Total SMMHC expression was decreased by 25% while the expression of NMM IIB (SMemb) was greatly increased by 4.5-fold. BLEB was found to completely relax detrusor strips from both sham-operated and PBOO rats pre-contracted with KCl, carbachol or electrical field stimulation although sensitivity was slightly decreased (20%) only at lower doses for PBOO. Thus we provide the first thorough characterization of the response of rat bladder myosin to PBOO and demonstrate complete BLEB-induced PBOO bladder SM relaxation. Furthermore, the present study provides valuable evidence that BLEB may be a novel type of potential therapeutic agent for regulation of myogenic and nerve-evoked DO in OAB.

## Introduction

Smooth muscle (SM) myosin (SMM) is the thick filament and motor molecule of the SM contractile apparatus, composed of a pair of myosin heavy chains (MHCs) and two pairs of myosin light chains (MLC_17_ and MLC_20_) that are intimately intertwined [1]. It has been shown that both the 3′ and 5′ end of the MHC pre-mRNA are alternatively spliced to generate COOH-terminal (SM1 and SM2) and NH_2_-terminal (SM-A and SM-B) isoforms, respectively. The SM-B isoform is predominantly found in SMs that demonstrate a more phasic contractile nature, faster shortening velocity and higher ATPase activity (e.g., urinary bladder, saphenous artery), whereas the SM-A isoform is found in slower more tonic-type SM with lower ATPase activity (e.g., aorta) [2]–[5]. Also, the essential light chain MLC_17_ is alternatively spliced and has two 3’ isoforms known as MLC_17a_ and MLC_17b_ [6], [7]. Similar to the SM-A and SM-B isoforms, the relative ratio of the MLC_17_ isoforms has been associated with the tonicity of SM with a higher ratio of MLC_17a_ to MLC_17b_ being associated with a more phasic type contraction [2], [8], [9].

Blebbistatin (BLEB) is a small cell permeable selective myosin II inhibitor that was originally discovered as the result of a high throughput screen for inhibitors of nonmuscle myosin (NMM) II [10]. Although originally thought to be much less efficacious on SM than NMMII, BLEB has now been suggested to inhibit SM contraction with near equipotency [11]–[13].

However, some data have suggested that BLEB is more efficacious at inhibiting SM tissues that express more SM-B SMM isoform. For example, Rhee et al. showed that force maintenance was inhibited by BLEB to a greater percent in bladder (mainly SM-B) than in aorta (mainly SM-A) while maximum bladder SM contraction was not altered but aortic SM was actually increased in the presence of BLEB [14]. In contrast, KCl-induced contraction of chicken gizzard (almost completely SM-B) was less potently (IC_50_ ∼20 µM) inhibited than the carotid artery that expresses predominantly SM-A (IC_50_ ∼3 µM) [11]. Thus, the influence of SM-A/SM-B splicing, which occurs very close to the BLEB binding site on the head of the myosin molecule and near the ATP cleavage site, is controversial.

In addition, it has been suggested that NMM II may contribute to tonic force maintenance [15]–[18]. Ekman et al. showed that BLEB was much more effective at inhibiting SM from neonatal vs adult bladder SM which expresses much lower levels of NMM II [16]. However, in contrast, Eddinger et al. showed that rabbit arterial SM was potently inhibited by BLEB (IC_50_ ∼5 µM) although this tissue does not express significant amounts of NMM II [11]. Thus, clearly the effect of SMM composition and relative amount of NMM II on BLEB efficacy also remains to be elucidated.

Most recently we provided novel data that BLEB also potently relaxed both rat and human bladder SM *in vitro* and rat detrusor *in vivo* and it was suggested that BLEB could be developed as a potential agent for overactive bladder (OAB) [13]. Partial bladder outlet obstruction (PBOO), a common urologic pathology mostly caused by benign prostatic hyperplasia (BPH), occurs in up to 70% of men over 60 years old [19]. However, OAB can coexist in 40–45% of patients with PBOO [20] and is associated with detrusor overactivity (DO) [21]. It has been documented that PBOO is associated with overexpression of SM-A and other SMM isoforms in obstructed animals with alteration in the expression of NMM II as well [22]. Concomitantly, the contractile characteristics of the bladder alter from a phasic to a more tonic-type contraction [22]–[26]. Thus, the aim of the current study was to investigate the effect of PBOO on rat bladder SMM isoform composition, NMM IIB expression and functional activities and to determine whether the effectiveness of BLEB is altered in the detrusor from PBOO rats.

## Materials and Methods

### Chemicals and tissues

All chemicals were from Sigma (St. Louis, USA) except (±) BLEB was from Tocris (Ellisville, MO, USA). The racemic mixture (±) of BLEB was used in all studies as it was determined that the active (-) enantiomer form was equipotent to the (±) racemic mixture in the *in vitro* studies and that the inactive (+) form did not induce significant relaxation [10], [11], [13], [27]. A stock solution of BLEB was made in dimethylsulphoxide (DMSO); the other substances were dissolved daily in double distilled water. Control experiments showed that the final concentrations (1/1000–3/1000) of DMSO used in these studies did not significantly modify the relaxation response induced by (±) BLEB. Due to the known light sensitivity of BLEB, it was always kept in the dark in the refrigerator until just prior to usage and during the experiment, the organ bath chambers were kept covered. Male rat urinary bladders were obtained from adult male Sprague-Dawley (SD) rats (Charles River; Raleigh, NC, USA). All animal studies were approved by the Animal Institute Committee of the Albert Einstein College of Medicine (study approval number 20100201).

### Partial bladder outlet obstruction model

As previously reported [28], [29], rats were anesthetized with pentobarbital (35 mg/kg) via an intraperitoneal (i.p) injection. A 2 cm midline vertical incision was made from the penoscrotal junction to the midscrotum to gain access to the bulbous urethra. The urethra was then isolated from the cavernous bodies. A sterile metal bar (19 gauge needle) with a 1.06 mm diameter was placed on the bulbous urethral surface, and a 3-0 polypropylene suture was used to place a tie around both the bulbous urethra and the bar. As soon as the suture was secured, the bar was removed, leaving the bulbous urethra partially obstructed. A 4-0 silk suture was used to reapproximate the muscle layer, and a 4-0 nylon suture was used to close the skin. Sham surgery was performed the same as described above except the urethral ties were not placed. All animals were kept 2 weeks on normal chow with a 12 h day/night light cycle.

### In vitro organ bath studies

The *in vitro* contractility studies were performed as previously described [12], [13], [30], [31]. Briefly, bladder dome strips containing urothelium with identical length were mounted longitudinally in a 4 ml Multi-Myograph Model 810MS (Danish Myo Technology; Aarhus, Denmark) organ bath. The myograph was connected in line to a PowerLab 4/30 Data Acquisition System (ADInstruments; Colorado Springs, CO, USA) and in turn to a Dual-Core processor Pentium computer for real-time monitoring of physiological force. The SM strips were equilibrated at least 1 h in Krebs-Henseleit (Krebs) buffer [12], [13], [28], [30], [31] at 37°C with continuous bubbling of 95% O_2_ and 5% CO_2_. The buffer had the following mM composition: NaCl 110, KCl 4.8, CaCl_2_ 2.5, MgSO_4_ 1.2, KH_2_PO_4_ 1.2, NaHCO_3_ 25 and dextrose 11 and it was changed every 15 minutes (min). Strips were continuously adjusted to 500–700 mg resting tension [32], [33]. After equilibration, rat detrusor was contracted with 60 mM KCl, cumulative concentrations (10^−8^–10^−4^ M) of carbachol or electrical field stimulation (EFS) at varying frequencies of 2, 4, 8, 16 and 32 Hz, duration 1.5 ms, train 5 s, and 80 V. Force produced by the above stimuli was normalized to strip weight. Next, strips were pre-contracted with 3 µM carbachol and allowed to reach stable tension and then the relaxant effects of increasing doses of BLEB were evaluated. Additionally, after pre-incubation with (±)BLEB (20 µM, equal to 10 µM active BLEB) for 30 min, its inhibitory effect on carbachol (3 µM) or aforementioned EFS mediated contractility was also tested.

### RNA extraction and cDNA synthesis

Total RNA was extracted using TRIzol reagent (Invitrogen; Carlsbad, CA, USA) according to the manufacturer's protocol. Briefly, the tissue was ground into a powder using a mortar and pestle cooled in liquid nitrogen without allowing the tissue to thaw. The powder then was homogenized immediately in denaturing buffer using a T8 Ultra-Turrax minielectric homogenizer (IKA Works; Wilmington, NC, USA), chloroform was added and mixed, the phases separated by centrifugation, and the RNA precipitated by isopropanol and then washed with 70% ethanol and dissolved in RNase-free sterile water. The resulting RNA was quantitated by spectrophotometry at 260/280 nm. Total RNA (1 µg) then was reverse transcribed using 0.5 µg oligo (dT)_12–18_ primer (Invitrogen), 500 µM dNTPs (Invitrogen), and 200 U of SuperScript II RNase H reverse transcriptase in a total volume of 20 µl for 50 min at 42°C.

### Competitive reverse transcriptase polymerase chain reaction (competitive RT-PCR)

As previously reported [34], polymerase chain reaction (PCR) was performed on 100 ng of the reverse transcribed cDNA using 2 units of Red Taq DNA polymerase (Sigma; St Louis, MO, USA), 200 ng each of upstream and downstream primer and 200 µM dNTPs (Invitrogen). SM-A/SM-B, SM1/SM2 and MLC_17a_/MLC_17b_ isoforms were amplified with competitive PCR, using a GeneAmp 9700 thermal cycler (Applied Biosystems; Foster City, CA, USA). The primer sequences are shown in Table 1. The cycling conditions were an initial 5 min at 94°C followed by 35 cycles (30 s at 94°C, 30 s at 55°C, and 120 s at 72°C), followed by a final one-time 7 min incubation at 72°C to ensure extension of all products.

**Table 1:** Primer sequences used to amplify target genes by PCR.

Target gene	Primer sequence (forward/reverse)
SM-A/-B	5′-AAGGCAAGAAAGACAGCAGCATCA-3′
	5′-TGCCGCCTCACATCTAT-3′
LC_17a/b_	5′-TGCATTGCCGAAAGCCTCCAG-3′
	5′-CAACATTCGACAGCTTTTGTCACT-3′
SM1/2	5′-GCTGGAAGAGGCCGAGGAGGAATC-3′
	5′-GAACCATCTGTGTTTTCAATAA-3′
MHC	5′-TTTGCCATTGAGGCCTTAGG-3′
	5′-GTTCACACGGCTGAGAATCCA-3′
NMM	5′-TGAGAAGCCGCCACACATC-3′
	5′-CACCCGTGCAAAGAATCGA-3′
RPL19	5′-GCGTCCTCCGCTGTGGTA-3′
	5′-CATTGGCGATTTCGTTGGT-3′

The PCR products were then separated by electrophoresis on a 2.5% agarose gel and were visualized using GelStar (Cambrex Bio Science Rockland, Inc.; Rockland, ME, USA) staining and ultraviolet illumination. Band density was quantified by reflectance scanning of gel photographs obtained with a BioDoc-It camera setup (UVP; Upland, CA, USA) using a Bio-Rad (Hercules, CA, USA) GS-700 imaging densitometer and subsequent analyses using the Bio-Rad Molecular Analyst 1D program that enabled us to obtain quantitative relative SMM isoform transcript expression data for all isoform pairs.

### Real-Time reverse transcriptase polymerase chain reaction (Real-Time RT-PCR)

As previously reported [31], RT products were also amplified in a 96-well plate in a 25 µl reaction volume with all samples run in triplicate, using the model 7300 Real-Time Thermocycler (Applied Biosystems). The following experimental protocol was utilized: denaturation (95°C for 10 min to activate the polymerase) followed by an amplification program repeated for 40 cycles (95°C for 15 s, then 60°C for 60 s) using a single fluorescence measurement. SMMHC and NMMHC targeted genes were amplified using SYBR Green for amplicon detection. For relative quantification, the efficiency of amplification for each individual primer pair (sequences shown in Table 1) was determined using cDNA target and the 2^-ΔΔct^ method [35] in conjunction with the RQ Study Software version 1.2.3 (Applied Biosystems). Gene expression was normalized to expression of the RPL19 ribosomal housekeeping gene.

### Statistical analysis

Results are expressed as mean ± SEM for n experiments. Statistical analysis was performed using either the Student's *t*-test (when two sample treatments were being compared) or using ANOVA when multiple means were compared. p<0.05 was considered significant.

## Results

Enlarged bladder mass was observed in the PBOO group with mean bladder weight significantly increased from 145.0±9.6 mg to 398.0±36.9 mg (P<0.001) representing a 2.74-fold increase. Since the body weight actually decreased in the PBOO rats, the bladder-to-body weight ratio actually increased 3.25-fold (Table 2).

**Table 2:** Rat body weight and bladder weight.

Group	Body wt (g)		Bladder wt (g)	%Bladder wt/Body wt
	Initial	Final		
Control (n = 14)	379.2±17.3	462.5±13.0	145.0±9.6	0.032±0.002
PBOO (n = 11)	392.3±14.1	372.5±19.6**	398.0±36.9**	0.104±0.011**

** = P <0.001 vs control.

**Figure 1:**
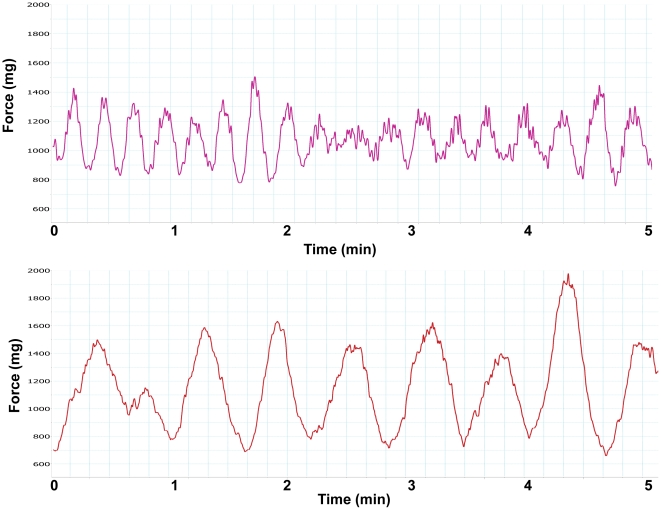
Typical tracings of rat urinary bladder strip spontaneous contractions. Stable spontaneous activity of rat detrusor strips was recorded. The x-axis represents time (min) while the y-axis represents force (mg). Upper panel is from the sham group while the lower panel is from the PBOO group.

**Figure 2:**
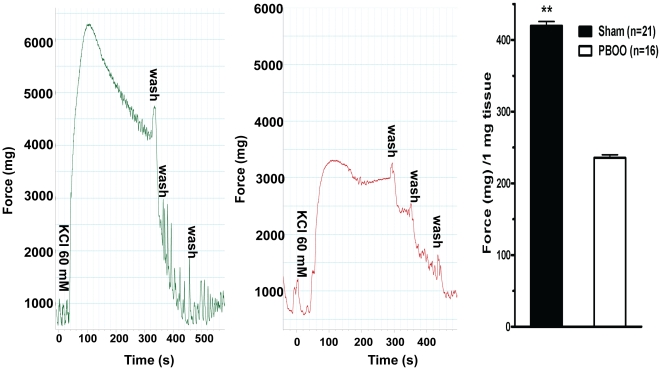
Rat bladder smooth muscle *in vitro* contractility in response to KCl. Left (sham) and middle (PBOO) panels are typical tracings. The x-axis represents time (s) while the y-axis represents force (mg). Accordingly, right panel is summary graph for the representative data shown in left and middle panels. Responses were normalized to strip weight. Values are expressed as mean ± SEM. ** = p<0.01 vs PBOO. (n  = strips obtained from 25 different animals, one to two strips were used for each animal).

**Figure 3 pone-0025958-g003:**
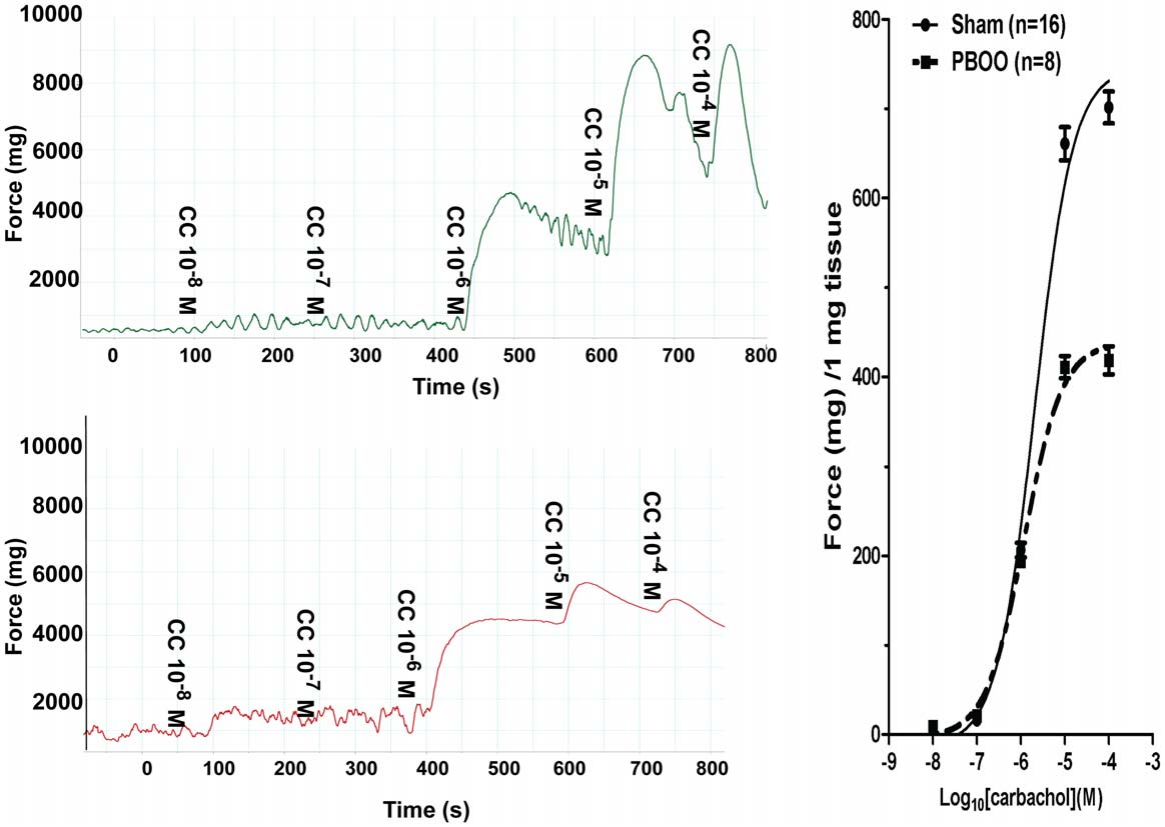
Rat bladder smooth muscle *in vitro* contractility in response to carbachol. Left panels are typical tracings. Upper portion of left panel is sham group while lower portion is PBOO group. The x-axis represents time (s) while the y-axis represents force (mg). Accordingly, right panel is summary graph for the representative data shown in left panels. Responses were normalized to strip weight. Values are expressed as mean ± SEM. (n =  strips obtained from 25 different animals, one to two strips were used for each animal).

**Figure 4:**
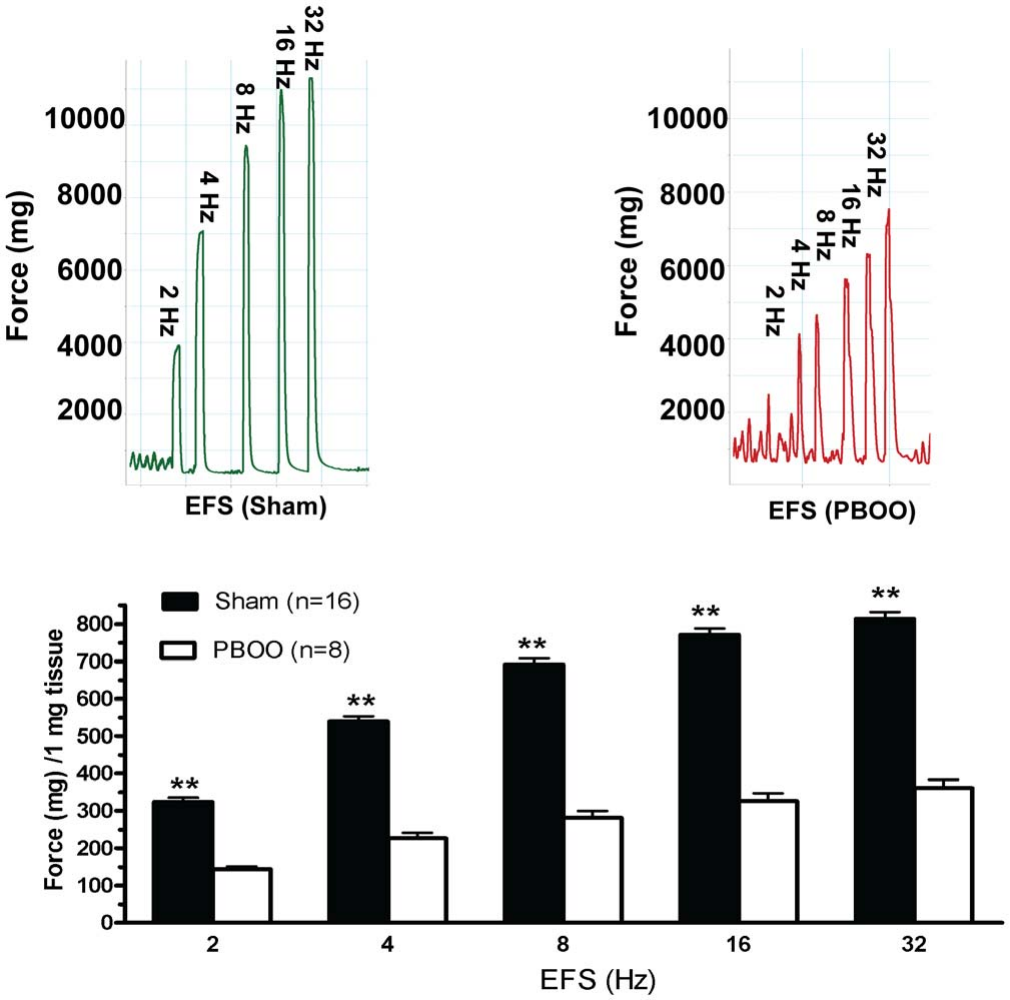
Rat bladder smooth muscle *in vitro* contractility in response to EFS. Upper panels are typical tracings. Left portion of upper panel is sham group while right portion is PBOO group. Accordingly, lower panel is summary graph for the representative data shown in left panels. Responses were normalized to strip weight. Values are expressed as mean ± SEM. ** = p<0.01 vs PBOO. (n  = strips obtained from 25 different animals, one to two strips were used for each animal).

Almost all bladder SM strips, after 30 min of equilibration at a resting tension of 500–700 mg displayed spontaneous contractions (Fig. 1). Obstruction altered detrusor spontaneous activities with contraction amplitude significantly increased (from 626 to 1191 mg force) but frequency significantly decreased (from 42.3 to 16.1 cycles/10 min) on average (Fig. 1, Table 3). PBOO also lessened bladder SM *in vitro* contractility and heightened tonicity. The force produced by bladder strips from PBOO rats was lowered by ∼50% in response to KCl depolarization (Fig. 2). For muscarinic receptor activation, ∼35% less contraction was observed at higher doses (10^−5^ and 10^−4^ M) of carbachol stimulation but there were no significant differences at lower concentrations (10^−8^ to 10^−6^) while EC_50s_ were similar at 2.09 and 1.19 µM for sham and PBOO, respectively (Fig. 3). PBOO was also associated with a 55–60% decrease in force generation in response to EFS at all stimulation frequencies (Fig. 4).

**Table 3:** Spontaneous activities of rat detrusor strips.

Group Frequency (cycles/10 min)		Amplitude (mg)
Control (n = 11)	42.3±3.3	626.0±118.1
PBOO (n = 9)	16.1±1.6**	1191.1±225.6*

* = P<0.01 vs control;

** = P<0.001 vs control.

**Figure 5:**
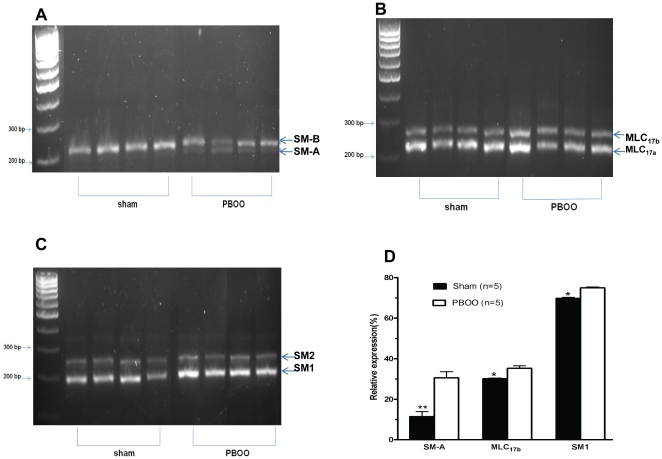
Rat detrusor smooth muscle myosin isoform composition. Panels A-C are representative GelStar-stained agarose gels of cDNA products resulting from competitive RT-PCR analysis of the SMMHC pre-mRNA of the 5’ region containing the SM-A/SM-B alternative splice site, the 17 kDa essential MLC pre-mRNA of the 3’ region containing the MLC_17a_/MLC_17b_ alternative splice site and the SMMHC pre-mRNA 3’ region containing the SM1/SM2 alternative splice site from sham and PBOO rats, respectively. Panel D shows averaged quantitative determination of SMM pre-mRNA mean isoform percentages in detrusor determined by using the information gathered from gels as in panels A–C. Values are expressed as mean ± SEM. * = p<0.05 vs PBOO; ** = p<0.01 vs PBOO. (n  = 5 different animals for each group).

PBOO bladder SM also exhibited a decreased shortening velocity as reflected by an approximately 2.5-fold slower time to 50% maximum contraction in response to both KCl and carbachol mediated contraction (27.9±8.3 vs 10.2±2.1 S and 35.0±8.0 vs 15.4±2.0 S, respectively)(Figs. 2 & 3) and much better maintenance of force. Consistent with elevated tonicity, obstruction altered bladder SMM isoform composition with the expression of SM-A relative to SM-B increased approximately 3-fold (from ∼10% to 30%) while MLC_17b_ (from 30% to 35%) and SM1 (from 70% to 75%) also increased significantly but to a lesser extent (Fig. 5).

**Figure 6:**
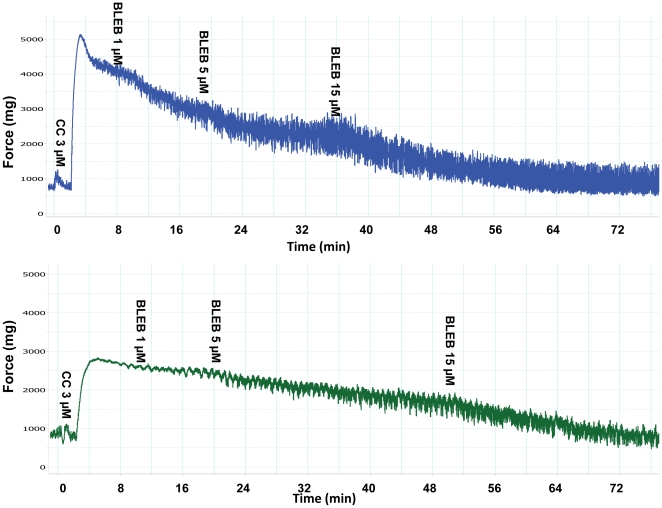
Representative tracings of BLEB-induced relaxation effects on detrusor SM pre-contracted with carbachol. Upper portion is the sham group while the lower portion is the PBOO group. The x-axis represents time (min) while the y-axis represents force (mg).

To determine whether the altered SMM isoform composition in the PBOO rat bladder influences BLEB inhibitory ability, various physiological experiments were performed. The results shown in Figs. 6 & 9 reveal that BLEB strongly and dose-dependently relaxed carbachol pre-contracted bladder SM strips from both sham and PBOO rats but exhibited ∼15% less efficacy in relaxing detrusor strips from PBOO rats at the lower doses of 1 µM and 5 µM but no difference was noted at 15 µM, a dose which totally attenuated the contraction of both preparations.

**Figure 7:**
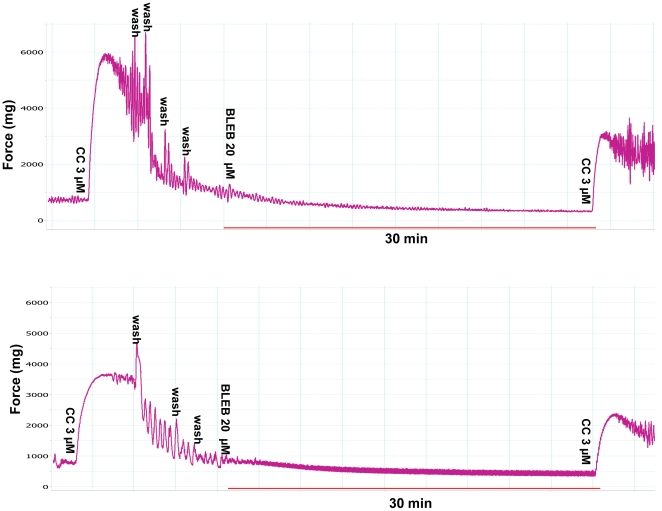
Representative tracings of BLEB-induced inhibitory effects on carbachol induced detrusor SM contractions. Upper portion is the sham group while the lower portion is the PBOO group. The x-axis represents time (min) while the y-axis represents force (mg).

**Figure 8:**
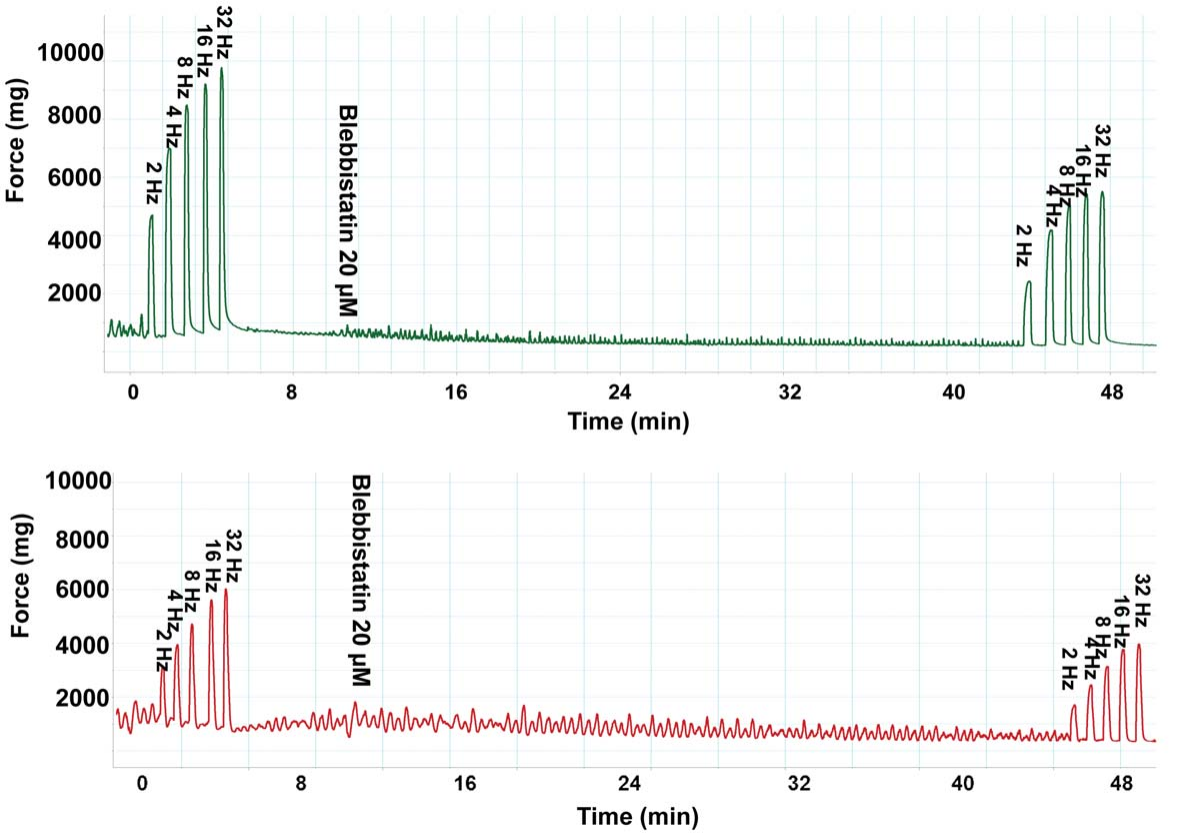
Representative tracings of BLEB-induced inhibitory effects on EFS induced detrusor SM contractions. Upper portion is the sham group while the lower portion is the PBOO group. The x-axis represents time (min) while the y-axis represents force (mg).

**Figure 9:**
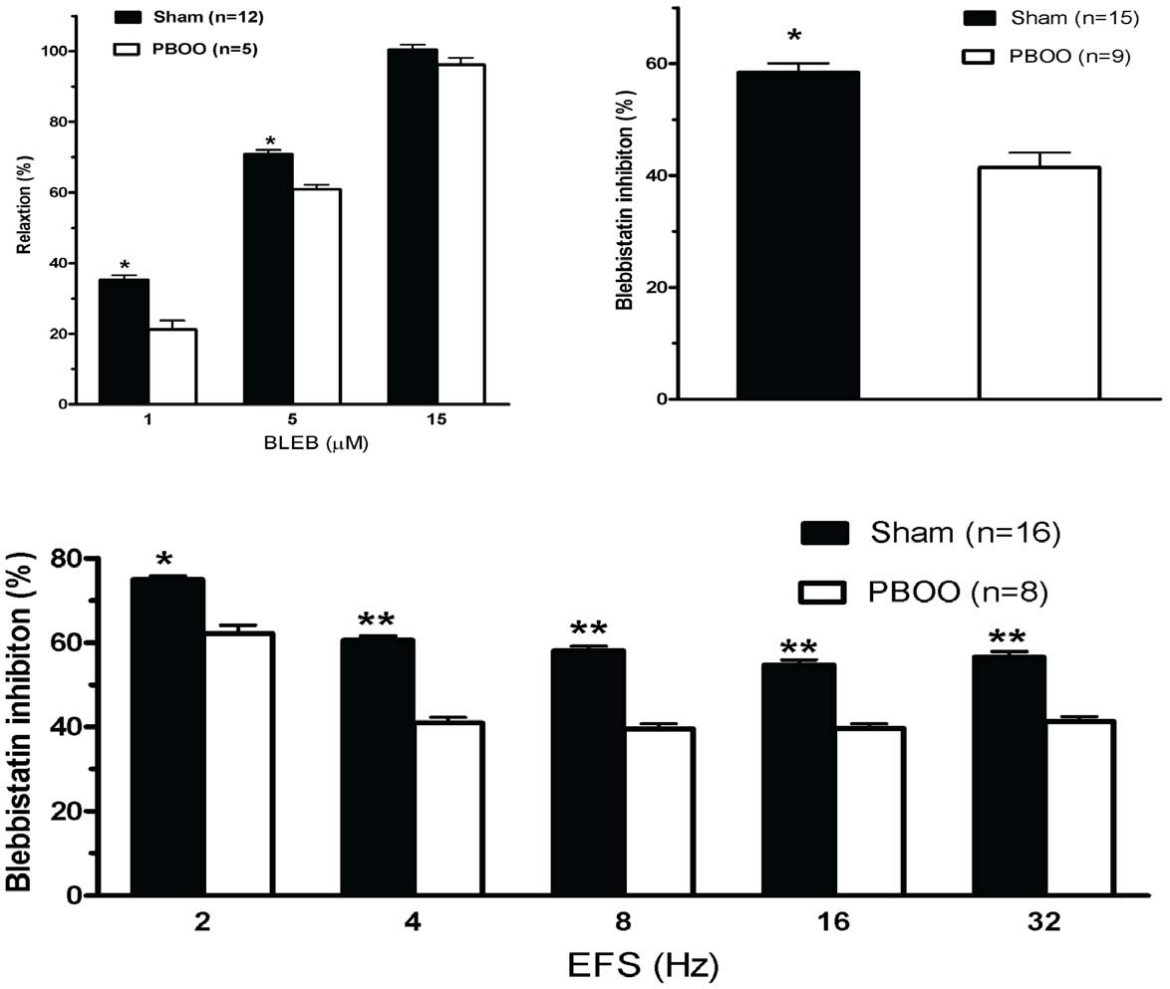
Summary graphs of BLEB relaxation and inhibitory effects on rat detrusor strip contractility. Upper left, upper right and lower panels summarize data shown in Fig. 6, 7 & 8, respectively. Response to stimulus was taken as 100%, while the relaxation or inhibitory effect of BLEB was evaluated as a percentage of this response. Values are expressed as mean ± SEM. * = p<0.05 vs PBOO; ** = p<0.01 vs PBOO. (n  = strips obtained from 25 different animals, one to two strips were used for each animal).

Preincubation of detrusor strips with 20 µM (±) BLEB (equal to 10 µM active BLEB) effectively inhibited 3 µM carbachol-induced tension increase for both sham and PBOO rats but inhibition was ∼20% less on detrusor from PBOO rats (Figs. 7 & 9). BLEB substantially inhibited EFS-induced contraction at all stimulation frequencies with inhibition at 2 Hz being more pronounced. When compared with sham, similarly, an approximately 20% lesser inhibition rate was observed frequency by frequency in PBOO rats (Figs. 8 & 9).

**Figure 10:**
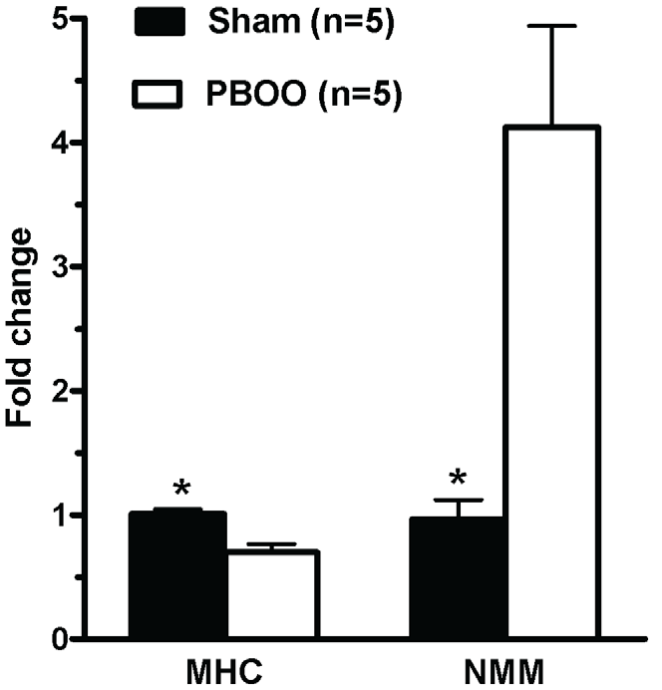
Expression of smooth muscle myosin heavy chain (SMMHC) and non muscle myosin heavy chain (NMMHC) in rat bladder. The studied molecules were quantified by Real-time RT-PCR. Expression is normalized to the RPL19 housekeeping gene. Values are expressed as mean ± SEM. * = p<0.05 vs PBOO (n  = 5 different animals for each group).

Finally, as some have suggested BLEB to be more efficacious on NMM II than SMM as described in the Introduction, we performed Real-Time PCR to quantify the relative expression of SMM II and NMM II. As demonstrated in Fig. 10, MHC transcript expression was significantly downregulated by approximately 30% while NMM expression was strongly upregulated ∼4.5- fold in obstructed bladder resulting in a more than 5-fold increase in the relative ratio of NMMHC II to SMMHC II transcripts.

## Discussion

The present study demonstrated that 2-week PBOO induced a significant relative overexpression of the SM-A and SM1 SMMHC isoforms as well as the MLC_17b_ essential light chain isoform in the rat bladder, which was associated with a switch to a more tonic-type SM contraction phenotype. Our novel data also revealed that the Type II myosin selective inhibitor BLEB potently relaxed detrusor SM, with sensitivity only slightly attenuated for detrusor from rats with PBOO but BLEB still able to provoke complete relaxation.

Our PBOO rat model was validated by a ∼3.5-fold increase in bladder weight within the 2-week obstruction period. It has been shown that PBOO can result in either a mild or severe obstruction depending upon factors such as the duration of obstruction [36] and the diameter of the surgical ligation [37]. In our study, we placed a 1.06 mm outer diameter 19 gauge needle on the bulbous urethral surface and tied the suture around the urethra with the needle bar similar to the technique used by Saito et al. to induce severe obstruction [37]. In the Saito et al. study, a ligation around a 1.09 mm catheter produced a 4-fold bladder weight increase close to our 3.5-fold increase, while an obstruction classified as mild was induced using a 1.70 mm outer diameter catheter and resulted in an average of only an approximately 80% increase in bladder weight.

Isolated detrusor strips of many small animals, including rats, often develop spontaneous activity, generally occurring upon action potential discharge resulting in Ca^2+^ influx through L-type Ca^2+^ channels and associated Ca^2+^ transients [38]. Our current data revealed that frequencies of spontaneous contractions in detrusors from sham rats outnumbered the ones from PBOO rats which became less phasic as stated above (Fig. 1 & Table 3). However, our data demonstrated that PBOO significantly heightened the amplitude of spontaneous contraction when compared to sham (Fig. 1 & Table 3). Yet, the exact mechanism underlying these pathophysiological changes remains to be ascertained.

As previously reported in male PBOO animals with more severe obstruction, bladder SM contractility was found altered in the present study with not only a switch to a more tonic-type contraction [22], [39], [40] but also a decrease in maximum force generation (35–50%) [25], [40]–[42] in response to various stimuli, including KCl depolarization, carbachol muscarinic receptor activation and EFS frequency-dependent intramural nerve activation (Figs. 2–4). Of note, at lower concentrations (10^−8^–10^−6^ M) of carbachol stimulation, contractile performance did not differ between the sham and PBOO group (Fig. 3), which may be attributed to increased sensitivity and an upregulation of M2 receptor expression in obstructed bladders to accommodate force loss as shown by other labs [43], [44].

It is likely that the loss of contractility may be associated with significant intracellular and extracellular changes: dysregulation of SM contractile protein expression, impaired energy production (mitochondrial dysfunction), calcium signaling abnormality, and increased detrusor collagen and relative denervation [45], [46]. However, SMM myosin isoform composition, the topic of our current study, is clearly important as transgenic knockdown of either SM-B [47] or SM2 [48] has been demonstrated to greatly alter bladder SM contractility. In fact, in the current study, obstruction for 2 weeks significantly increased the mRNA level of SM-A 3-fold from 10% to 30% (Figs. 5A & D), paralleling the aforementioned more tonic-type bladder SM contraction and previous studies in other species [22], [25]. Also our approximate 50% decreased contraction in the PBOO rat bladder correlates well with the lessened isometric detrusor force generation by almost 50% in the SM-B isoform knockout mice [47]. Moreover, to our knowledge, our current study is the first report on the effect of PBOO on the relative expression of the SM-A and SM-B alternatively spliced SMM isoform pair in the rat.

In addition to the attenuated contraction, the time to reach 50% maximal contraction was increased by PBOO for both KCl and carbachol stimulation at 27.9±8.3 vs 10.2±2.1 S and 35.0±8.0 vs 15.4±2.0 S, respectively, as can be seen in Figs. 2 & 3. The deceased shortening velocity of the detrusor from PBOO rats correlated with the slower shortening velocity measured in aorta (expressing mainly SM-A) compared to saphenous artery (expressing mainly SM-B) [5] and the decreased shortening velocity of bladder SM from SM-B knockout compared to wild type mice [47], [49]. Hence, the switch to a greater SM-A isoform composition for PBOO bladder is related to a decreased force generation and shortening velocity resembling a more tonic SM phenotype.

We further characterized the 3’ alternatively spliced SM1/SM2 and the essential light chain isoforms. The relative expression of the MLC_17b_ mRNA was also increased but only by about 5% from 30% to 35% (Figs. 5B & D). An increase in the detrusor MLC_17b_ isoform was similarly reported in response to rabbit PBOO. However, the relative expression of MLC_17b_ in the normal rabbit detrusor was only ∼8% but then increased more than 7-fold to ∼37% in response to PBOO, a value similar to that of the rat PBOO detrusor [22]. In mammalian SM tissues, the relative expression of the MLC_17b_ isoform has been found to exhibit an inverse relationship with the Vmax of the particular SM tissue [50] and MLC_17_ isoform exchange/reconstitution demonstrated that the relative MLC_17b_ expression related to a lower ATPase activity and Vmax [9], [51], [52] thus correlating with our current finding in the rat PBOO model. Again, the SM1 mRNA was increased by only about 5% compared to SM2 mRNA from 70% to 75% (Figs. 5C & D). This is similar to what has been reported in the rabbit and mouse in response to PBOO but the changes in these species were much greater with the rabbit SM1 increased from ∼35% to ∼50% [24], [26] and mouse SM1 increased from 47% to 80% [25]. Of note, the 70% relative expression of SM1 is much higher in the normal rat bladder than either the rabbit or mouse.

It has been reported that the tail of the SM1 isoform may inhibit contraction [53], [54] which would support a role for the increased SM1 expression correlating with our decreased force generated by detrusor from PBOO rats. However, selective knock out of the SM2 isoform (thus all SM1 type MHC remaining) in mice has been shown to increase KCl and carbachol-induced contraction [48] and transgene overexpression of SM1 in mice increased bladder contraction by 92% while in contrast transgene overexpression of SM2 decreased bladder contraction by 80% [55]. Thus, based upon these two latter SM1/SM2 MHC genetic manipulation experiments, the increased expression of SM1 in the current study would actually be expected to increase force rather than to decrease.

Since it has been suggested that BLEB efficacy may be impacted by the relative expression of the SM-A/SM-B SMMHC isoform composition, we compared the efficacy of BLEB at inhibiting bladder SM from sham rats to that from PBOO animals which we demonstrated to have an altered SMM isoform composition including a 3-fold increase in the relative expression of the SM-A isoform. Our data revealed that the myosin Type II selective inhibitor BLEB potently relaxed carbachol pre-contracted detrusor SM force maintenance and preincubation with BLEB attenuated carbachol and EFS induced detrusor contraction, with sensitivity only slightly attenuated for detrusor from rats with PBOO but with BLEB still able to provoke complete relaxation of the PBOO bladder strips. It has been reported that the PBOO/BPH bladder has enhanced permeability resulting from distension or inflammation [56]. Thus, it is unlikely the urothelium of PBOO rats may blunt the BLEB effect.

Finally, to further explore the fact that detrusor strips from PBOO rats was slightly less responsiveness to BLEB, the expression of SMMHC II and NMMHC II were examined with Real-Time RT-PCR. Our data revealed that SMMHC transcript expression decreased by 30% while NMMHC transcript expression increased by ∼4.5-fold (Fig. 10). As BLEB has been found not to compete with ATP binding or inhibit myosin light chain kinase [10] or alter SMM regulatory light chain phosphorylation levels[14], it thus appears that BLEB functions via binding to the myosin-ADP-P_i_ complex and blocking the myosin II in an actin-detached state. Therefore, the 30% downregulation of SMMHC and/or the more than 5-fold increase in the relative ratio of NMMHC II to SMMHC II possibly contributed to the ∼20% decreased effect of BLEB for SM from PBOO rats.

However, BLEB was found still effective for PBOO strips. As shown in Figs. 6 & 9,15 µM (±)BLEB (equal to 7.5 µM active BLEB) totally and equally relaxed bladder preparations for both groups. Moreover, BLEB can directly relax prostatic SM *in vitro* (data not show). Since BPH is a common illness coexisting in 40–45% of patients with OAB [20] which remains in 20–30% patients even after surgery [57], it is important to note that BLEB may be a potential therapeutic agent for these patients. As BLEB relaxes SM via a novel targeting of the SM contractile apparatus, it is further suggested that when intravesically delivered, side effects such as the bothersome dry mouth related to anticholingeric therapy can be avoided. However, we have difficulty to keep BLEB in the PBOO bladder long enough and *in vivo* experimentation was not performed in the present study. Therefore, further studies are required. It is important to find a practical BLEB delivery approach for the PBOO/OAB model and BLEB dosing should be carefully determined with attention paid to find a dose which relieves OAB symptoms without impairing voiding contraction. Another limitation for the current study is that protein levels of SMM isoforms were not determined, as SM-A/SM-B antibodies are not commercially available at present. However, our previous study in partial outlet obstructed rabbit bladder demonstrated that mRNA level correlated well with protein expression [22].

In conclusion, bladder outflow obstruction altered detrusor SMM isoform composition, expression and functional activities. PBOO bladder SM contractility was attenuated and switched from a phasic-type SM to a more tonic phenotype with overexpression of SM-A, MLC_17b_, SM1 and NMM. Our novel data also showed that the myosin II selective inhibitor BLEB potently inhibited bladder SM contraction but its efficacy decreased slightly with obstruction but only at lower doses. Importantly, it seemed that the SMMHC played a more important role than NMMHC in modulating BLEB inhibitory ability. The present study provides valuable evidence that BLEB may be a potential therapeutic agent for the regulation of myogenic and nerve-evoked DO in OAB.
